# Integration of diffusion tensor imaging parameters with mesh morphing for in-depth analysis of brain white matter fibre tracts

**DOI:** 10.1093/braincomms/fcae027

**Published:** 2024-02-22

**Authors:** Maryam Tayebi, Eryn Kwon, Jerome Maller, Josh McGeown, Miriam Scadeng, Miao Qiao, Alan Wang, Poul Nielsen, Justin Fernandez, Samantha Holdsworth, Vickie Shim, Matai mTBI Research Group, Leigh Potter, Paul Condron, Davidson Taylor, Daniel Cornfield, Patrick McHugh, Taylor Emsden, Helen Danesh-Meyer, Gil Newburn, Graeme Bydder

**Affiliations:** Auckland Bioengineering Institute, The University of Auckland, Auckland, 1010, New Zealand; Mātai Medical Research Institute, Gisborne, 4010, New Zealand; Auckland Bioengineering Institute, The University of Auckland, Auckland, 1010, New Zealand; Mātai Medical Research Institute, Gisborne, 4010, New Zealand; GE Healthcare, Richmond, Victoria, 3122, Australia; Mātai Medical Research Institute, Gisborne, 4010, New Zealand; Mātai Medical Research Institute, Gisborne, 4010, New Zealand; Faculty of Medical and Health Sciences, The University of Auckland, Auckland, 1023, New Zealand; Department of Computer Science, The University of Auckland, Auckland, 1010, New Zealand; Auckland Bioengineering Institute, The University of Auckland, Auckland, 1010, New Zealand; Faculty of Medical and Health Sciences, The University of Auckland, Auckland, 1023, New Zealand; Auckland Bioengineering Institute, The University of Auckland, Auckland, 1010, New Zealand; Department of Engineering Science, The University of Auckland, Auckland, 1010, New Zealand; Auckland Bioengineering Institute, The University of Auckland, Auckland, 1010, New Zealand; Mātai Medical Research Institute, Gisborne, 4010, New Zealand; Department of Engineering Science, The University of Auckland, Auckland, 1010, New Zealand; Mātai Medical Research Institute, Gisborne, 4010, New Zealand; Faculty of Medical and Health Sciences, The University of Auckland, Auckland, 1023, New Zealand; Auckland Bioengineering Institute, The University of Auckland, Auckland, 1010, New Zealand; Mātai Medical Research Institute, Gisborne, 4010, New Zealand

**Keywords:** DTI, data visualization, PCA, free form deformation, statistical morphometry

## Abstract

Averaging is commonly used for data reduction/aggregation to analyse high-dimensional MRI data, but this often leads to information loss. To address this issue, we developed a novel technique that integrates diffusion tensor metrics along the whole volume of the fibre bundle using a 3D mesh-morphing technique coupled with principal component analysis for delineating case and control groups. Brain diffusion tensor MRI scans of high school rugby union players (*n* = 30, age 16–18) were acquired on a 3 T MRI before and after the sports season. A non-contact sport athlete cohort with matching demographics (*n* = 12) was also scanned. The utility of the new method in detecting differences in diffusion tensor metrics of the right corticospinal tract between contact and non-contact sport athletes was explored. The first step was to run automated tractography on each subject’s native space. A template model of the right corticospinal tract was generated and morphed into each subject’s native shape and space, matching individual geometry and diffusion metric distributions with minimal information loss. The common dimension of the 20 480 diffusion metrics allowed further data aggregation using principal component analysis to cluster the case and control groups as well as visualization of diffusion metric statistics (mean, ±2 SD). Our approach of analysing the whole volume of white matter tracts led to a clear delineation between the rugby and control cohort, which was not possible with the traditional averaging method. Moreover, our approach accounts for the individual subject’s variations in diffusion tensor metrics to visualize group differences in quantitative MR data. This approach may benefit future prediction models based on other quantitative MRI methods.

## Teaser

Data reduction methods such as averaging are commonly used in high-dimensional MRI analysis, but this results in information loss. However, individual variability makes group-wise comparisons difficult without averaging. Tayebi *et al*. introduces a novel method that combines 3D mesh-morphing techniques with principal component analysis that allows MRI metric group comparisons without averaging.

## Graphical Abstract

**Graphical Abstract:**
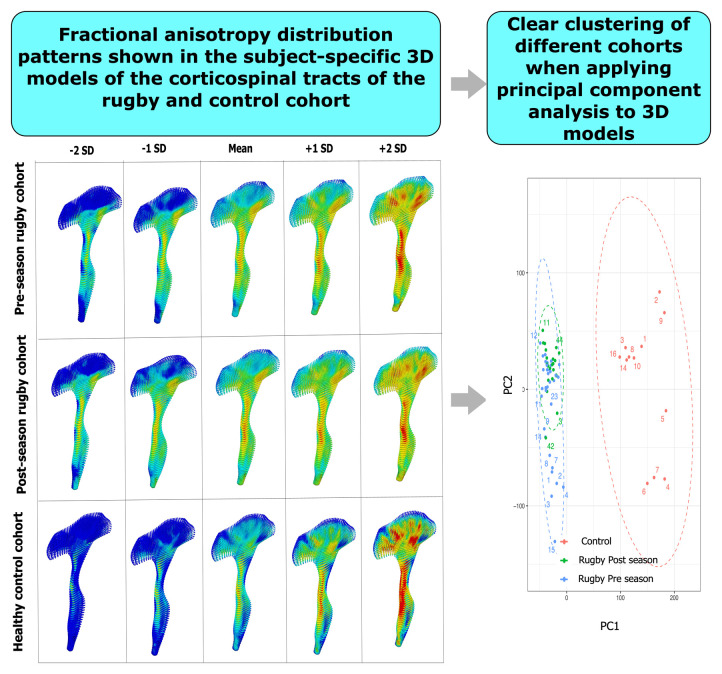

## Introduction

Current advancements in MRI technology have resulted in a significant shift from qualitative techniques to quantitative methods, allowing quantifiable measurements of tissue’s physical or physiological characteristics in scalar units. These new approaches in MRI techniques increase the sensitivity and specificity of the final diagnosis.^1^ However, this shift has led to a considerable rise in the number of quantitative measurements from various MR sequences. Dealing with such high-dimensional data is becoming of increasing concern for scientists in the imaging field. This ‘curse of dimensionality’^2^ applies in particular to neuroimaging, where multiple imaging sequences are often acquired—with each sequence often outputting multiple metrics or maps—making it challenging to analyse and visualize such multi-dimensional data.

Dimensionality reduction is one solution for this problem. Here, the number of variables required to describe the original high-dimensional data is reduced. Feature extraction, one main category of dimensionality reduction, downgrades the complexity of original data by providing a new smaller set of features that preserve essential information.^3^ For example, principal component analysis (PCA) reduces the number of dimensions according to the variance within the data.^4,5^ Applying PCA to reduce multi-dimensional MR image data can increase the computational efficiency of machine-learning algorithms for identifying patterns of degeneration or injury and improve the overall predictive performance.^6^

Diffusion tensor imaging (DTI) is a key quantitative MR sequence used in neuroimaging applications that can benefit from using PCA. By measuring the microscopic mobility of water molecules, DTI provides insights into the microstructure of brain tissue.^7,8^ Since the diffusion direction of the water molecules is largely influenced by the organized structure of the white matter (WM) fibre tracts, DTI can describe the directionality of the brain fibre tracts.^9^ Several quantitative (scalar) images can be extracted from the DTI sequence, including the degree of diffusion anisotropy [fractional anisotropy (FA)], axial diffusivity (AD), radial diffusivity (RD) and mean diffusivity (MD) at each imaging voxel. Furthermore, based on local estimates of the principal diffusion direction, DTI allows for identifying underlying WM trajectories in the brain tissue, known as tractography.^10,11^ Tractography is routinely used as a non-invasive and qualitative method of reconstructing and visualizing brain fibre tracts in both clinical and research settings.

Currently, there are multiple methods of merging these vector (tractography) and scalar (diffusion metrics) measurements. The conventional approach is to calculate the average diffusion metric (FA, MD, AD or RD) as a single scalar value over the entire length of each fibre bundle.^12^ A newer method, called along-tract profiling, is able to measure these scalar values at multiple nodes along each fibre tract.^13^ In this method, each fibre bundle is resampled to a number of equally spaced nodes (varied between 20 and 100) along the central portion of the tract, and diffusion properties are summarized at each node by taking a weighted average of the diffusion properties.^12^ A more popular tool called Tract-Based Spatial Statistics (TBSS) is an improved version of the conventional voxel-based analysis technique.^14^ In this method, each participant’s generated diffusion metric map will be projected onto a skeleton located at the centre of major WM bundles.^15^

The major limitation of the methods above is the loss of information due to averaging. Summarizing the diffusion metrics over a whole fibre bundle to one or a few scalar values makes it hard to capture important variations that might present along the tract, especially in the orthogonal directions to the main tract skeletons. Even the TBSS skeletonization technique reduces an entire volume of WM bundle to a single voxel thick sheet.^16^ Increasing the spatial resolution of the image will also pose a higher risk of information loss during the projection steps. However, these limitations can be mitigated by measuring the diffusion indices along the whole volume of the fibre bundles to minimize information loss. This study aims to develop such a method by embedding DTI metrics inside a 3D mesh, which is then morphed to match the subject’s geometric and diffusion tensor information using a well-known mesh-morphing method.^17^ PCA is then used to characterize the patterns and variations in the DTI metrics along the whole volume of the fibre tract.

To demonstrate the predictive power of this method for distinguishing a particular cohort from a control group, we apply this approach to multi-metric DTI scans of rugby union players to determine if subtle changes in their brain after repetitive head impacts can be detected. Contact sport athletes are of particular interest as they are often subjected to multiple repetitive impacts. Investigating the changes in the brains of contact sport players provides a means to minimize confounding variables as the frequency and extent of the head impacts are measured and quantified. This in turn will allow us to compare the impact-induced changes with age-matched non-contact sport athletes.^18^ In the literature, studies utilizing diffusion MRI have identified regions of the brain that are most susceptible to head injuries among contact sport athletes, and the corticospinal tract consistently stands out as one of the most sensitive WM pathways.^19^ Given its evident vulnerability, we decided to start our analysis from this tract.

## Materials and methods

### Participants

Thirty male rugby players in the age range of 14–18 years participated in this study. To be included in the study, participants were required to be at least 14 years of age, speak English as their native language and have no contraindications to MRI. Excluded players included those with a history of concussion within the last 6 months before the study, a severe brain injury or other neurological deficits or pathology. Players underwent brain scans at two time points: before starting the rugby season and after the season. The number of players who were scanned at post-season reduced to 20; hence, we used those 20 players whom we have both pre- and post-season scans. Twelve age-matched non-contact sport male athletes were recruited for the control group for a single timepoint scan. All participants involved provided written informed consent prior to study participation. This study was approved by the New Zealand Health and Disability Ethics Committee (20/NTB/14), and all participants involved provided written informed consent prior to study participation.

### Accelerometer mouthguard

A bespoke kinematic sensor mouthguard, HitIQ® Nexus A9, was custom fitted to rugby players to measure repetitive head impacts, recording linear and angular head accelerations during games and practices. This involved using a NI9205 analogue input module with a sample rate of 20 kHz, 16 bit and stored using a LABVIEW program.^20^ Data were processed to ascertain acceleration at the head’s centre of mass using the NAP algorithm^21^ and then compared to accelerometer readings to detect errors. Impact data underwent pre-processing to eliminate readings with prediction values below 0.9, based on the proprietary HitIQ® algorithm. This ensured the removal of dubious impacts, with the refined data reflecting only genuine impacts. Accumulated linear and angular accelerations were calculated for each player during the entire rugby season to get an estimate of their impact load.

### MRI acquisitions

Brain MRI scans from both groups were acquired using a 3 T MRI scanner (SIGNA Premier, General Electric Healthcare, Milwaukee, WI; AIR™ 48-channel head coil). A 3D T_1_-weighted IR-prep, fast IR-SPGR (inversion recovery RF-spoiled gradient echo) sequence (TR (Repetition Time) = 6.6 ms, TE (Echo Time) = 2.7 ms, flip angle = 12°, TI (Inversion Time) = 600 ms, matrix size = 512 × 512, FOV (Field of View) = 224 mm × 224 mm, number of slices = 320, voxel size = 0.4 mm × 0.4 mm × 0.5 mm) was acquired and used as a reference for image registration. A multi-shell spin echo diffusion-weighted MRI was also performed (TR = 4500 ms, TE = 70 ms, matrix size = 128 × 128, FOV = 260 mm × 260 mm, number of slices = 80, voxel size = 2 mm × 2 mm × 2 mm, parallel reduction factor = 2, *b*-value = 0, 1000, 2000 and 3000 s/mm^2^, 54 gradient directions = 4, 15, 15 and 20, respectively) to provide structural connectivity of the brain.

### MR image processing

All image processing steps were performed using FSL (FMRIB software library; http://fsl.fmrib.ox.ac.uk/fsl/, version 6.0).^22-24^

#### Diffusion MR processing

Diffusion MR images were processed using FDT (FMRIB’s Diffusion Toolbox) as part of the FSL software package.^25^ Diffusion images were initially pre-processed by running a topup function for estimating the susceptibility-induced off-resonance field.^23,26^ The outputs from topup were fed into the eddy tool, which corrects eddy current–induced distortions and subject movements.^27^ DTIFIT was then applied to reconstruct a diffusion tensor. This tool locally fits a diffusion tensor model at each voxel of the diffusion image and generates 3D tensor maps, including FA, MD, AD and RD maps.

#### Automated tractography

Tractography was done using TractSeg, an openly available automated tract segmentation tool.^28^ This tool is a novel, fully convolutional neural network–based approach that is fast and highly accurate and does not need additional registration or parcellation techniques.

In order to estimate the fibre orientation distribution, the multi-shell, multi-tissue constrained spherical deconvolution technique from Mrtrix^29^ was used. The principal directions were then extracted from the fields of fibre orientation distribution function peaks with a maximum number of three peaks per voxel. The result of tractography is 72 fibre tracts, and right corticospinal tract (R-CST) was used for further analysis, which is commonly implicated in head injuries sustained during contact sports.^19^

### Embedding DTI metrics in a 3D mesh via nonlinear morphing

An existing high-fidelity 3D template mesh of the R-CST comprised of hexahedral elements was used to generate a subject-specific 3D model of the R-CST using the subject’s MRI. First, the MRI segmentations were exported as surface models. This was then used to morph the template mesh to the subject’s geometry using free-form deformation (FFD). Note that we have used the latter method extensively in the past in subject-specific finite element model generation for the brain^30,31^ and other soft tissues.^32,33^

**Figure 1:**
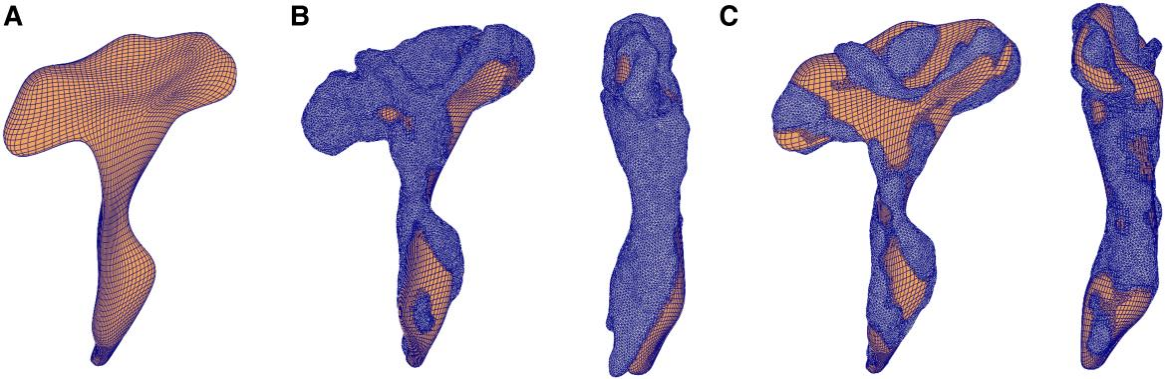
**Subject-specific mesh-morphing procedure.** FFD process that morphs a template mesh to a new subject’s geometry. (**A**) Template mesh created from a subject roughly resembles the generic shape of the R-CST (coronal view). (**B**) A total of 20 480 squares are overlaid on each specific subject’s geometry (left: coronal, right: sagittal). Since the match is not optimized for each subject, the template mesh is deformed to minimize the distance between data points and the surface of the mesh. (**C**) The optimized subject-specific fitted mesh (left: coronal, right: sagittal). Both coronal and sagittal views of R-CST are shown for **B** and **C**.

The benefit of using the FFD method is that the relative distances between the nodes in the 3D model are preserved as the morphing is done by embedding a mesh to be customized (in our case, the 3D mesh of the RCST) inside a wrapper mesh. Hence, the nodes on the external surfaces are fitted to the cloud of data points from the MRI segmentation of the subject, while the internal nodes were transformed using the same deformation as the external nodes. This enabled us to perform inter-subject comparisons with the resulting subject-specific models. This was done to all subjects’ MRI data, resulting in a total of 42 3D subject-specific models of R-CST for both rugby and control cohorts. Figure 1 describes the overall FFD procedure applied to the R-CST.

Once the 3D model was fitted to the subject’s geometry, both the 3D model and the MR images were aligned with each other in the same space. This allowed us to link each node (hence element) in the model with the nearest voxel in the MRI space using the nearest neighbour search algorithm in Python (Scipy spatial cKDTree). This was successfully used in our previous works on finite element analysis of mild traumatic brain injury (mTBI).^30,31^

**Figure 2:**
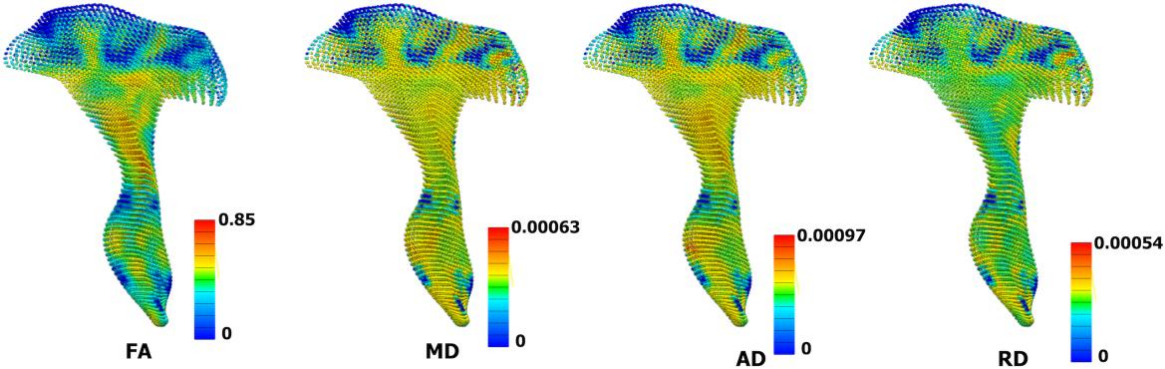
**3D meshes are assigned with four different DTI metrics (coronal view).** Each metric has different scales, as shown in the colour bars next to the model. FA, fractional anisotropy; MD, mean diffusivity; AD, axial diffusivity; RD, radial diffusivity.

The code for performing this is in the open-source Python library called gias 3, which is available at https://github.com/musculoskeletal/gias.^3^ Using this code, the MRI DTI indices (FA, MD, RD and AD) are stored as field information for each element, which was later used in the PCA analysis (Fig. 2).

Since a single template mesh was used to generate all subsequent subject-specific models, every subject’s model had the same number of elements and nodes, which is essential in performing the PCA analysis described below.

### Statistical analysis

#### PCA

PCA was used to linearly transform the diffusion parameters from each element of the 3D model into a new lower-dimension feature space such that the variance (the largest eigenvectors of the correlation matrix) of the data is preserved. We used the ‘pcaMethods’ library from R statistical software (version 4.1.2) to perform PCA. Specifically, it was conducted on the extracted diffusion metrics from the R-CST [four diffusion parameters (FA, MD, AD and RD) for each element (total number of elements = 20 480)] to generate independent, linear combinations for each individual. The input matrix was centred and scaled prior to PCA to ensure the diffusion parameters were comparable to each other and preserve the variance between individuals. PCA components 1 and 2 were then used to visualize the clustering of healthy (non-athlete) controls versus cases (contact sport players) based on the individual variance in the brain region of interest (R-CST).

#### TBSS

In the study, TBSS was used to perform voxel-based analysis comparing diffusion metrics between two distinct groups. Specifically, separate comparisons were conducted between the pre-season group and a control group, as well as between the post-season group and the same control group. TBSS was carefully applied across all diffusion metrics, including FA, MD, AD and RD. This approach aimed to highlight significant statistical differences between the groups in question. To strengthen the reliability of the analysis, a high number of random permutations, set at 5000, along with threshold-free cluster enhancement was used. This combination effectively controlled the family-wise error rate, ensuring that the chance of any type I errors stayed below the accepted significance level of *P* < 0.05. This method provided clear insights into the microstructural differences in WM tracts between the groups studied.

## Results

**Figure 3:**
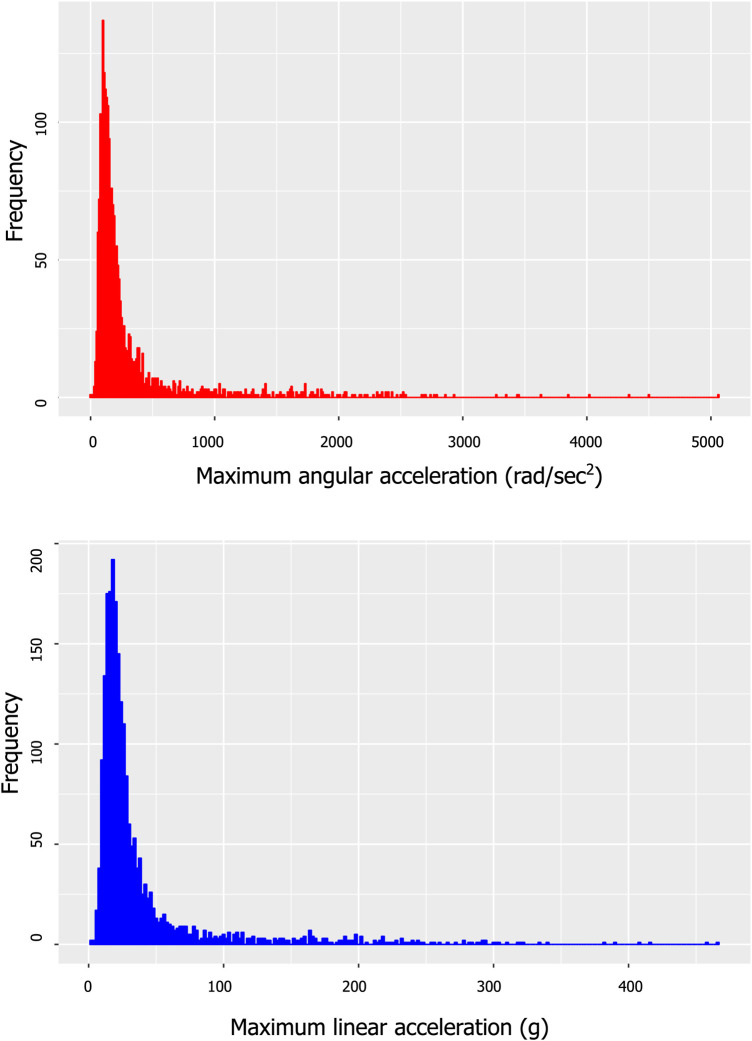
**Distribution of head impacts that the players in the rugby cohort received throughout the season.** The graph on the left show impacts in terms of linear accelerations while the graph on the right shows impacts in angular accelerations.

Out of the initial 30 rugby players, 20 participated in the post-season scan. As a result, we incorporated only those individuals with dual scans into our analysis. The athletes in this group played an average of 6.8 ± 4 games. The head impacts that they experience during games and practices were measured with instrumented mouthguard sensors (HitIQ Nexus A9), which were moulded to fit the oral cavity of each player in the rugby cohort. None of the players experienced significant head impacts (e.g. concussion) or any other mTBI events throughout the season. The distribution of head impacts (both in terms of linear and angular accelerations) that the players received throughout the season is given in Fig. 3.

### TBSS analysis

**Figure 4:**
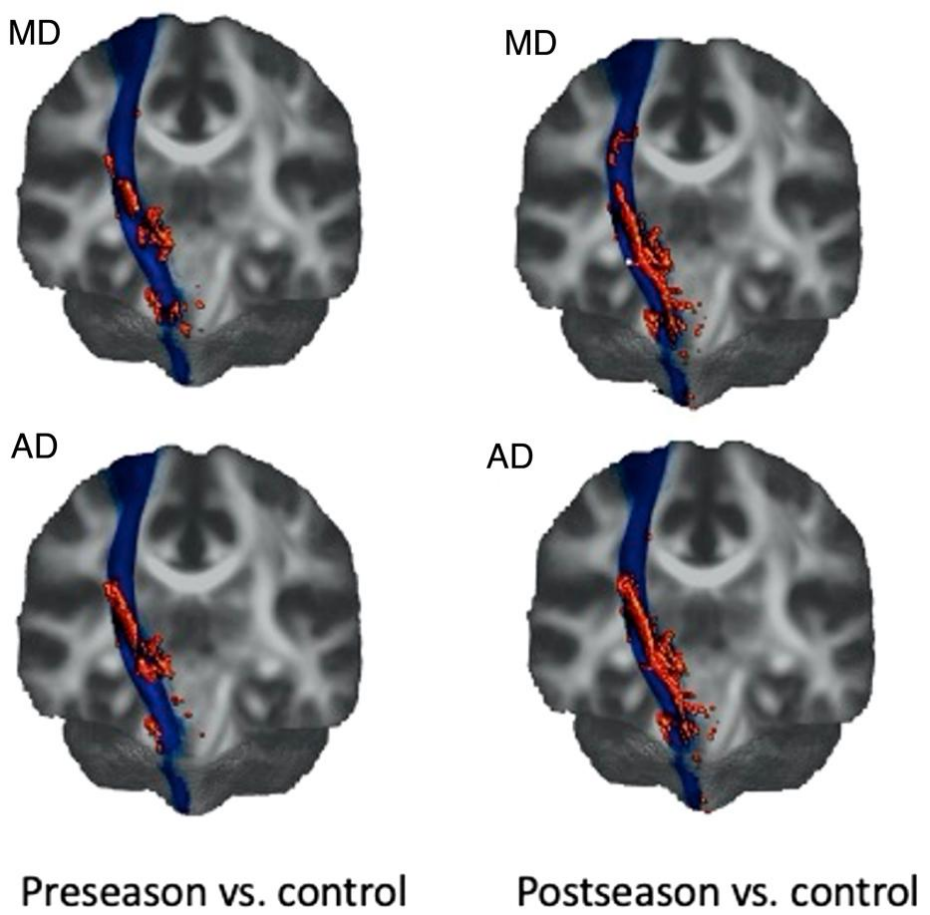
**3D rendering of TBSS results from R-CST overlaid on the mean FA (coronal view).** The central line area shows the R-CST extracted from the mean FA skeleton using the R-CST mask of JHU ICBM-DTI-81 WM atlas. The dispersed spheres and shapes around the central line show TBSS results of statistically significant difference between pre-season and control (left)/post-season and control (right) for each respective diffusion metric. Only the results from MD and AD are shown, as FA and RD showed no change. MD, mean diffusivity; AD, axial diffusivity.

The two groups’ unpaired *t*-test was performed on pre-season scans of rugby players versus the control group and post-season versus control athletes. Since the main focus of the current study is on the R-CST, Fig. 4 demonstrates the areas on the R-CST where significant differences between groups were found. Compared to the non-contact sport athletes, rugby players showed significant differences in MD and AD (no change in FA and RD) along the R-CST. However, post-season scans depicted larger areas of difference in MD and AD (a 106 and 63% increase compared to pre-season, respectively).

### PCA performed with 3D models

The mesh morphing combined with the nearest neighbour search algorithm generated subject-specific 3D models that match the subject-specific shape of the R-CST (with the average root mean squares error <1 mm), while each element in the mesh was assigned with the corresponding DTI metrics.

**Figure 5:**
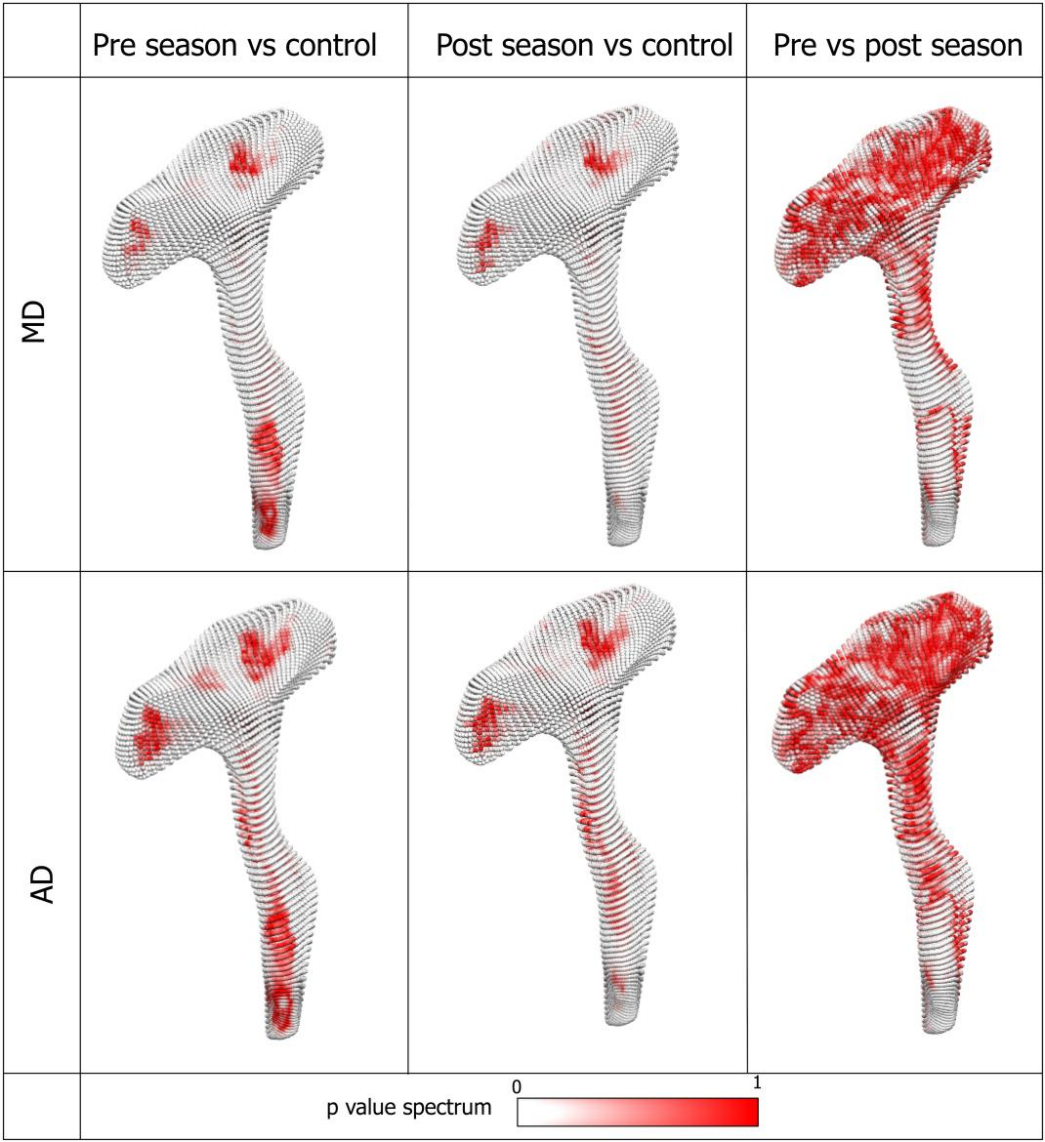
**Element-wise *t*-test between rugby and control cohorts as well as pre-post comparison within the rugby cohort.** The result is displayed as a distribution of the *P*-values in a red and white spectrum. The white indicates areas with smaller *P*-values (hence significant difference), and the red indicates higher *P*-values (hence less significant difference). When compared with the control cohort, more elements become significantly different at post-season as indicated by the increase in the white regions in the CST tract (the first two columns). When the pre- and post-season comparison was made, the extent in the area showing difference is much smaller as shown by the large areas with red elements. MD, mean diffusivity; AD, axial diffusivity.

First, we performed the element-wise statistical *t*-test on our 3D models to see how DTI metric values of each element are significantly different between rugby and control cohort. Figure 5 shows that locations and distributions of the significantly different element when compared with the control cohort are similar to TBSS findings in Fig. 4. The number of elements with significant difference increased at post-season, especially around the spine of the CST tract (Fig. 5). On the other hand, when pre- and post-season results are compared, the number with significant differences decreased as can be seen in the widening red area in the last column of Fig. 5.

These 3D meshes embedded with DTI metrics were then used in the PCA analysis. Specifically, all four diffusion variables (FA, MD, AD and RD) were assigned as a field variable in the corresponding node of the 3D mesh, allowing us to investigate the effects of variation of these values with relation to its 3D location within the CST. Each feature was standardized by rescaling the features such that they have the properties of a standard normal distribution with a mean of zero and a SD of 1. Then, all diffusion indices were reduced to their first two principal components

**Figure 6:**
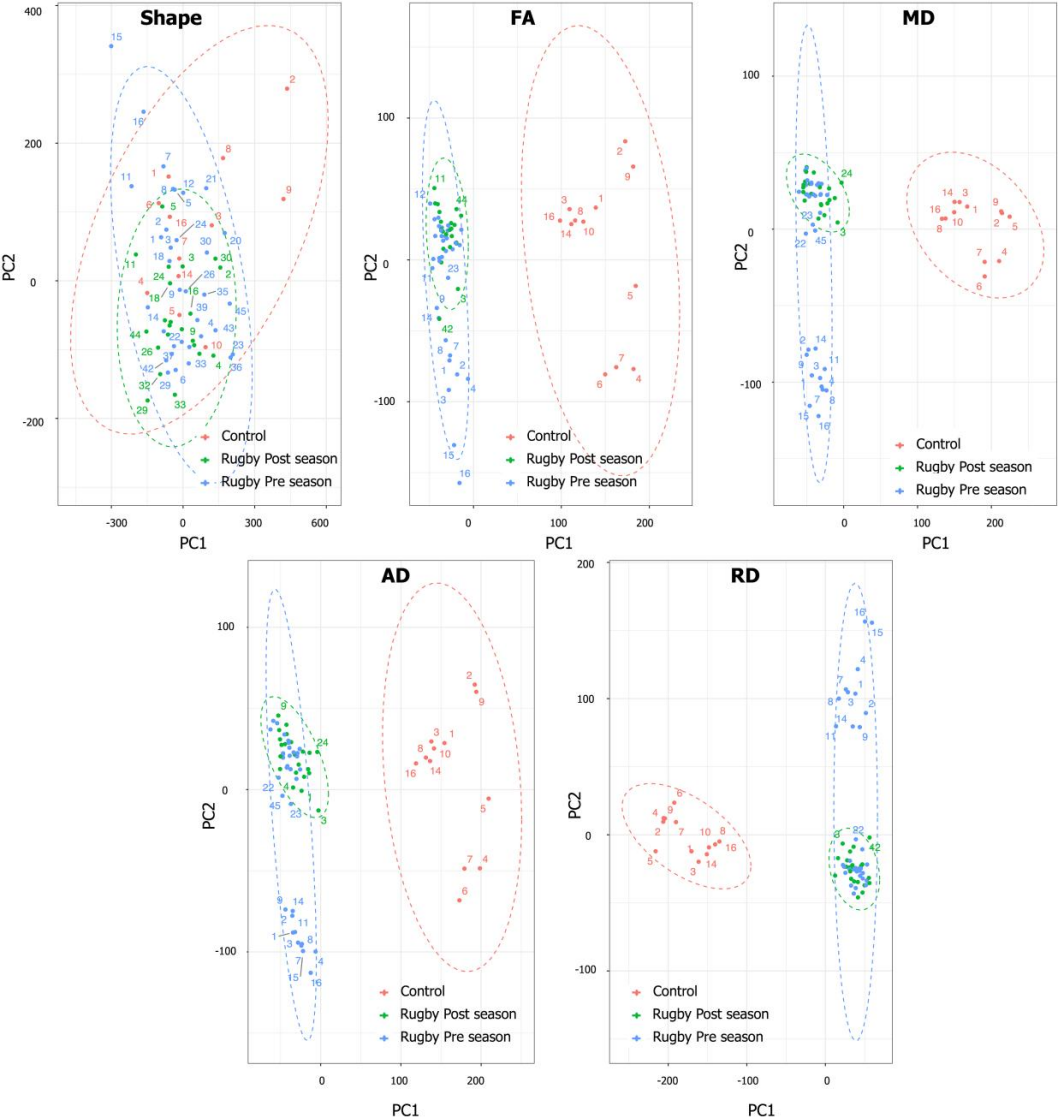
**Results of PCA. PC1 and PC2 show the clustering of the case (pre-season and post-season) and control for each diffusion metric separately: FA, MD, AD, RD and the shape.** FA, fractional anisotropy; MD, mean diffusivity; AD, axial diffusivity; RD, radial diffusivity.

Clustering the PCA data [PC1 and PC2, accounting from 33% (FA) to 47% (RD) of the data variation] allowed for discrimination between rugby players versus control cohorts (non-contact sport playing athletes) based on the major variance in the data (DTI measurements from each node of the R-CST model; Fig. 6).

Interestingly, when only the shape of the tract was considered, the principal component plots (PC1 and PC2) did not show any clustering between the cohorts (Fig. 6). However, the principal component plots (PC1 and PC2) built from DTI metrics showed distinguishable clusters for both pre- and post-season scans against the control cohort for all four DTI metrics. This is in contrast to the TBSS results, where only MD and AD showed the difference between the pre-season rugby cohort and the control cohort. This indicates that incorporating orthogonal components into the skeleton of the tract as well as their geometrical location improves the discernibility of DTI metrics. Furthermore, we observe some degree of clustering within the rugby cohort between pre- and post-season scans, where MD and AD display more discernible clustering within the rugby cohorts at the post-season. This result is consistent with TBSS results where the differences grew more prominent for MD and AD at the post-season.

**Figure 7:**
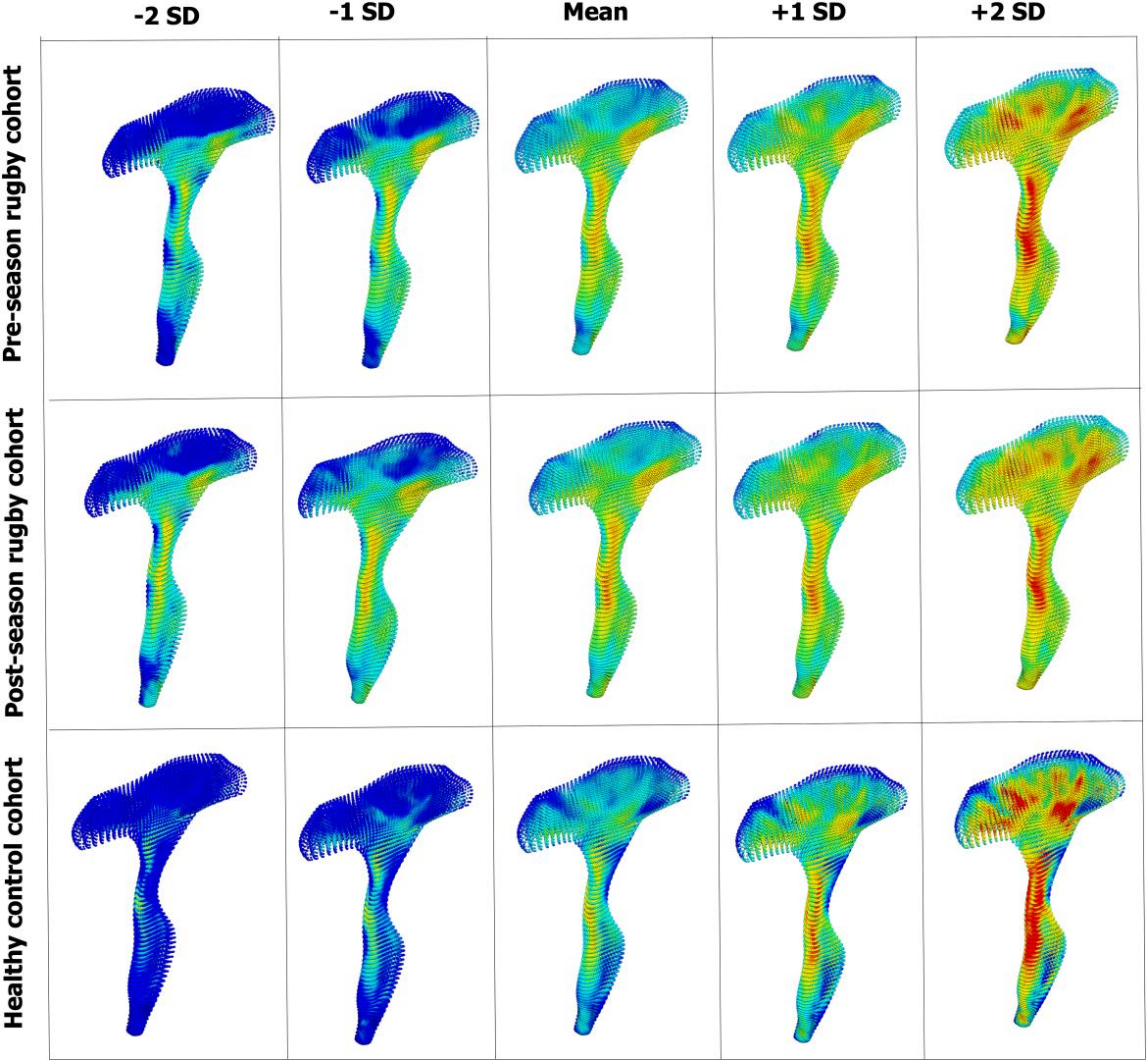
**The pattern of FA value distribution in three different cohorts.** Different patterns of variations are visible, especially between the rugby and control cohorts, indicating the changes in the DTI metrics from subconcussive impacts. The colour scale is the same as Fig. 2 (0∼0.85).

We also compared the 3D distribution patterns of DTI metrics among the three cohorts over the entire CST tract (Fig. 6, central column). We used FA, in which TBSS found no significant differences between control and rugby cohorts. The 3D distributions, however, display distinctive patterns between these cohorts. The rugby cohorts (both pre- and post-season) display more evenly spread FA distributions than the control cohorts, indicating that the repetitive head impacts might have differential effects on different regions of the CST, which is not apparent when considering an average over the entire tract. Moreover, we can see the difference between pre- and post-season cohorts where the high FA regions in the central part become more pronounced after the season, which also indicates that location-dependent changes in the diffusion metrics have occurred after repetitive head impacts. The changes within the cohort were also examined by comparing the mean distribution patterns with other patterns at ±1 and ±2 SD. As can be seen from Fig. 7, the changes within the cohort are much more pronounced in the control cohort, especially in the superior region of the tract, than the rugby cohort. Overall, this result highlights the need to consider the changes in the entire tract rather than averaging them into a single or a few different values over a skeleton. Distribution patterns of other DTI metrics (MD, AD and RD) are given in Supplementary Figs 1–3, respectively.

## Discussion

The main goal of this project was to introduce a novel approach to investigating the brain’s WM tracts that combine different techniques from different disciplines—DTI analysis from MRI, mesh morphing from computer graphics and PCA from statistical data analysis. We showed that by first generating the 3D template model of the CST (one of the most vulnerable tracts in the brain to head injury) and morphing it into the subject’s native space, we were able to make a subject-specific model of the CST tract that matched both the geometry and DTI index distribution of the subject. This led to the development of subject-specific 3D models consisting of the same number of nodes and elements for all subjects, each carrying multiple scalar values as field information within the nodes. Importantly, our model provides 20 480 registered locations in the R-CST that are matched across all participants. This data structure allows for downstream analysis, such as dimensionality reduction and case–control clustering.

The application of our method on DTI scans of contact sport players displayed apparent clustering of contact sport and non-contact sport athletes. It is worth noting that there is no correlation between PC1 and PC2 because they are orthogonal (independent) to each other, even though they are used to classify controls and cases.

Moreover, having the equivalent template mesh morphed into a subject-specific data set means that we could visualize the pattern of changes that occur within the entire WM tract. This new knowledge will help identify those who exhibit an abnormal pattern as being at risk for developing persistent symptoms. Further analysis with a bigger data set will show the feasibility of this approach.

Within the last decade, the increasing number of multimodal MRI studies has led to a higher demand for analysis techniques that can deal with such high-dimensional data. Magnetization transfer imaging,^34^ T_1_/T_2_/T_2_* mapping,^35,36^ quantitative susceptibility mapping^37^ and diffusion MRI^38^ are some examples of such quantitative techniques that have been widely used by scientists in various fields of medical research, which result in high-dimensional data sets.

DTI, which is the main focus of our study, provides multiple numerical variables. The most common diffusion indices, FA, MD, AD and RD, have been extensively used in the studies of brain tissue microstructural changes such as neurodegenerative disease,^38-40^ brain injury,^41,42^ cancer^43,44^ and aging.^45,46^

When studying the brain, there are two different approaches to conducting the group analysis: inference and prediction techniques.^47^ Many empirical studies use inference methods to detect differences that report *P*-value as a sign of a statistically significant difference between groups. Voxel-based analysis techniques^48^ and connectometry^49^ are categorized as inference methods.

In contrast, the prediction approach tries to recognize potentially meaningful patterns in the study groups,^50^ which are often ‘found’ rather than obtained from carefully planned experimental studies.^47^ Given the explosion of high-dimensional big data sets in recent years, one of the keys to achieving the required prediction accuracy is to build a quantitative model incorporating multi-dimensional data.^51^ A successful prediction algorithm is able to identify subtle patterns in a big data set. Having a data set with rich information that can correctly define the population is crucial to increase the accuracy of the predictive model.

In this regard, we focused on four features from the diffusion MR sequence that have the potential to generate >10 different features on a large scale. To increase the accuracy of the predictive model applied to the data extracted from DTI, we need to acquire this information from as many data points as possible from the participant’s scan. The current techniques available are limited to the averaging method, such that the whole data points were averaged to a single value or multiple-segmented (up to 100) values.

Studies that conduct prediction analysis using DTI data on groups of participants with various diseases apply the tract-profiling technique to extract multiple diffusion indices along the specific tract of interest. The details of the profilometry method are well explained by Yeatman *et al*.^12^ In summary, tract profiling resamples the whole tract bundle to 20–100 equally space nodes located in the centre of the bundle. The diffusion metrics corresponding to each vertex are then projected and averaged along each bundle segment, weighted according to its distance from the bundle core. However, averaging the whole bundle of tracts with thousands of data points to 100 scalar values will clearly lead to a non-trivial information loss. Our proposed method can overcome this limitation by including as many nodes as possible with <10% loss of information along the tract. This means our model is able to provide a precise informative map regarding the metric of interest along the entire WM fibre tract, which allows us to run predictive analysis successfully.

Finding reliable imaging biomarkers to help scientists quantitatively analyse subtle changes that occur in the brain due to early stages of disease or injury is a challenging topic in research. Imaging biomarkers for detecting concussion (mTBI) in high-risk sports are particularly challenging.^19^ Studies comparing the brain of contact sport and non-contact sport players have presented conflicting results due to the heterogeneity in the analysis methods, diffusion sequences and study population.^52,53^ Despite this, the CST has commonly been identified as one of the most vulnerable tracts to multiple head impacts, particularly among athletes who play contact sports^54^; thus, it was chosen in this work as the first region of interest to undergo model generation. By applying our method to all four extracted diffusion metrics (FA, MD, AD and RD) from the R-CST, differences in diffusion variables were found between age- and sex-matched contact sport and non-contact sport athletes. The inherent ability of PCA to visualize clusters makes this technique a potential tool to detect structural abnormalities in the brain of patients with mTBI and other conditions.

Our approach is not to analyse whether certain DTI metrics increased or decreased after playing contact sport. Rather, we provide a new approach that embeds DTI metrics inside the 3D deformable model, which led to the clear delineation between a rugby and control cohort, which was not possible with traditional averaging approaches. Moreover, the DTI metrics are embedded in every node and element of the model, which allows one to analyse the combined effects of these four DTI indices in the future as well, which, to our knowledge, has not been possible in the past.

Feature extraction techniques have been used in many areas, such as neurological and psychiatric disorders,^55^ developing brain^56^ and amyotrophic lateral sclerosis.^57^ However, these studies mainly applied single averaging or tract profiling to extract the diffusion indices. To the best of our knowledge, no studies have applied dimensionality reduction techniques on the combined diffusion imaging metrics and shape to distinguish between two groups, in our case contact sport players versus non-contact sport athletes’ brains. By combining our novel method and PCA, we demonstrated that each diffusion index embedded inside the 3D model could distinguish contact athletes and non-contact sport players. This finding highlights the importance of considering DTI metrics in terms of its 3D locations and feature extraction techniques. By finding the variables involved in PC1 and PC2, we will be able to run a correlation test between these PCs and other clinical or neurocognitive variables, which has the potential to be a robust predictive algorithm for identifying the effects of subtle changes in the brain more broadly. Moreover, the fact that we were able to achieve clear clustering with relatively small cohorts (especially the control cohort) gives us confidence that the additional data that we are currently collecting will provide more avenues of exploration of this method such as expanding to other tracts and incorporating non-MRI variables such as brain strain patterns.

There are some limitations in our study. We acknowledge the absence of definitive ground truth for discerning subtle microstructural alterations in the brain following repetitive head impacts. Various approaches are being developed to enhance the precision of detecting these changes; however, ongoing research in this domain necessitates further time to establish robust experimental validation.

Our analyses revealed changes in the R-CST between the rugby group’s pre-season and post-season compared to controls. As all participants were adolescents, some changes could be attributed to natural brain development. Future research will adjust for age effects by scanning controls at intervals equivalent to a rugby season. Additionally, given the 6-month gap between scans in rugby cohort, signal drift could be another limitation. In this study, standard pre-processing steps have been applied to the diffusion scans to mitigate this; however, it is important to note that signal drifting is an emerging concern that needs consideration. Signal drift particularly in echo-planar imaging sequence (e.g. diffusion MRI) refers to the potential inconsistencies in scanner performance over time,^58^ which can introduce variability in the acquired data, potentially affecting the accuracy of longitudinal comparisons. Therefore, caution is required when interpreting our results.

To dissect the R-CST tract, we used TractSeg. This tool is designed to automatically segment and label tracts from diffusion MRI data. In some cases, the tool might mislabel or miss tracts due to noise, artefacts or anatomical variations. However, we applied standard pre-processing pipeline to decrease the risk of these factors.

Using mesh-morphing techniques for CST reconstruction in the brain has advantages but comes with limitations. These include potential inaccuracies due to complex CST geometry and anatomical variability, challenges with accurately capturing fibre crossings and curvature, potential mesh distortion and artefacts, reliance on template mesh resolution, lack of clear ground truth for validation, computational intensity, parameter sensitivity and the need for user expertise in pre-processing and interpretation.

## Conclusion

In conclusion, our novel method has the ability to extract the diffusion metrics at registered locations with the least amount of information loss. It provides sufficient data that can be fed into the prediction model. We showed the applicability of our model in separating diffusion metrics in contact sport athletes and non-contact sport players. Further work will include running this method on a bigger database to train the prediction model to improve concussion (mTBI) diagnosis. Applying our mesh-morphing method with feature extraction techniques will let scientists conduct predictive studies that integrate multiple quantitative variables from multimodal MRI and apply it in various fields of neuroimaging research

## Supplementary Material

fcae027_Supplementary_Data

## Data Availability

Original MRI data are not available on request due to ethical and legal obligations to research participants. The MRI diffusion analysis and mesh-morphing pipeline are available upon request from the authors.

## Appendix I

### Mātai mTBI Research Group

Leigh Potter, Paul Condron, Davidson Taylor, Daniel Cornfield, Patrick McHugh, Taylor Emsden, Helen Danesh-Meyer, Gil Newburn, and Graeme Bydder
