# Supplementary material for: Integration of diffusion tensor imaging parameters with mesh morphing for in-depth analysis of brain white matter fibre tracts
Source: Brain Commun. 2024 Feb 22;6(2):fcae027. doi: 10.1093/braincomms/fcae027 (PMC11024816; doi:10.1093/braincomms/fcae027)
Supplement: fcae027_Supplementary_Data [file fcae027_Supplementary_Data.docx]

**
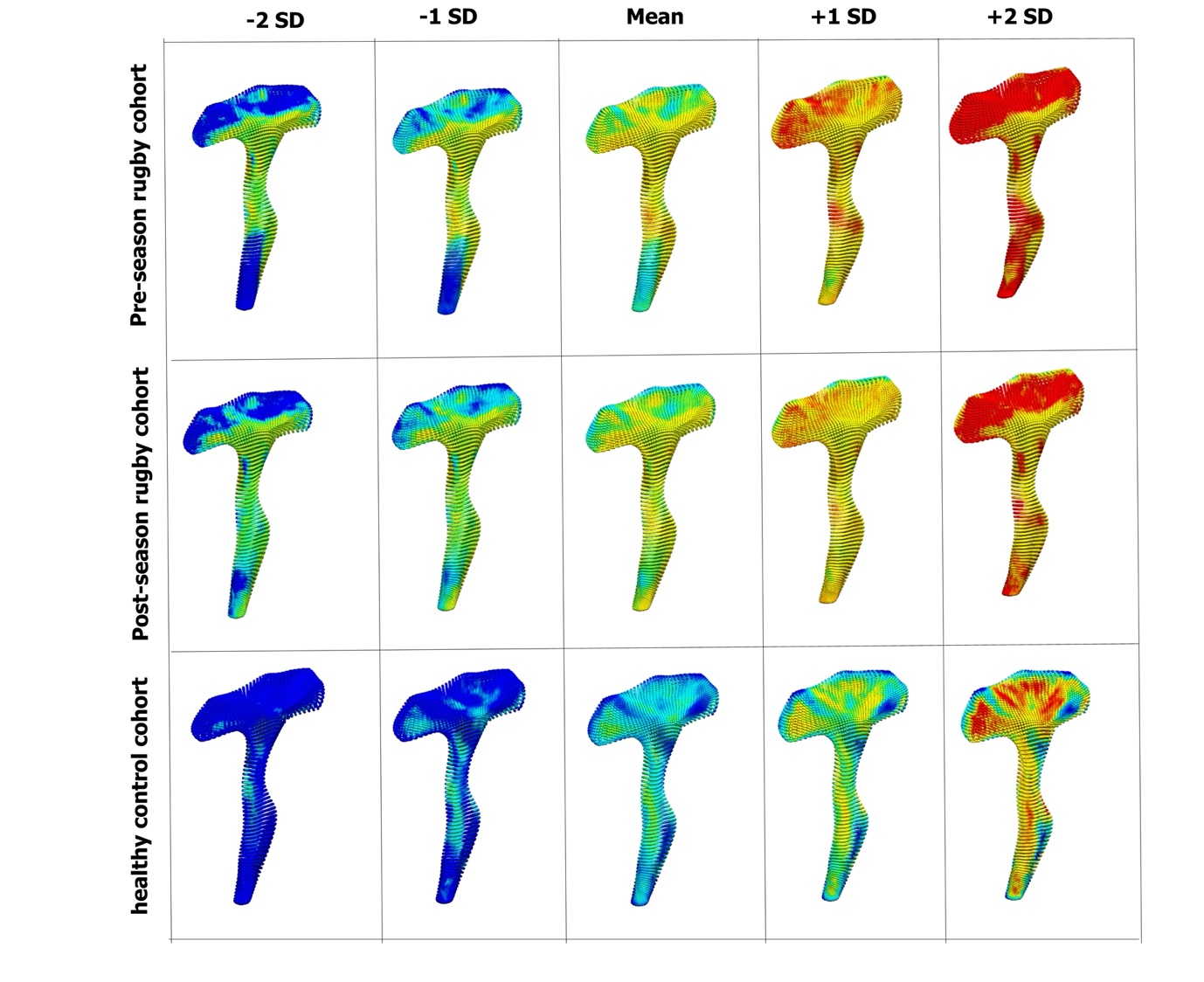
Supplementary Figure 1:** Pattern of MD value distribution in three different cohorts. The colour scale is the same as Figure 2 in the manuscript (0 ~ 0.00063)

**
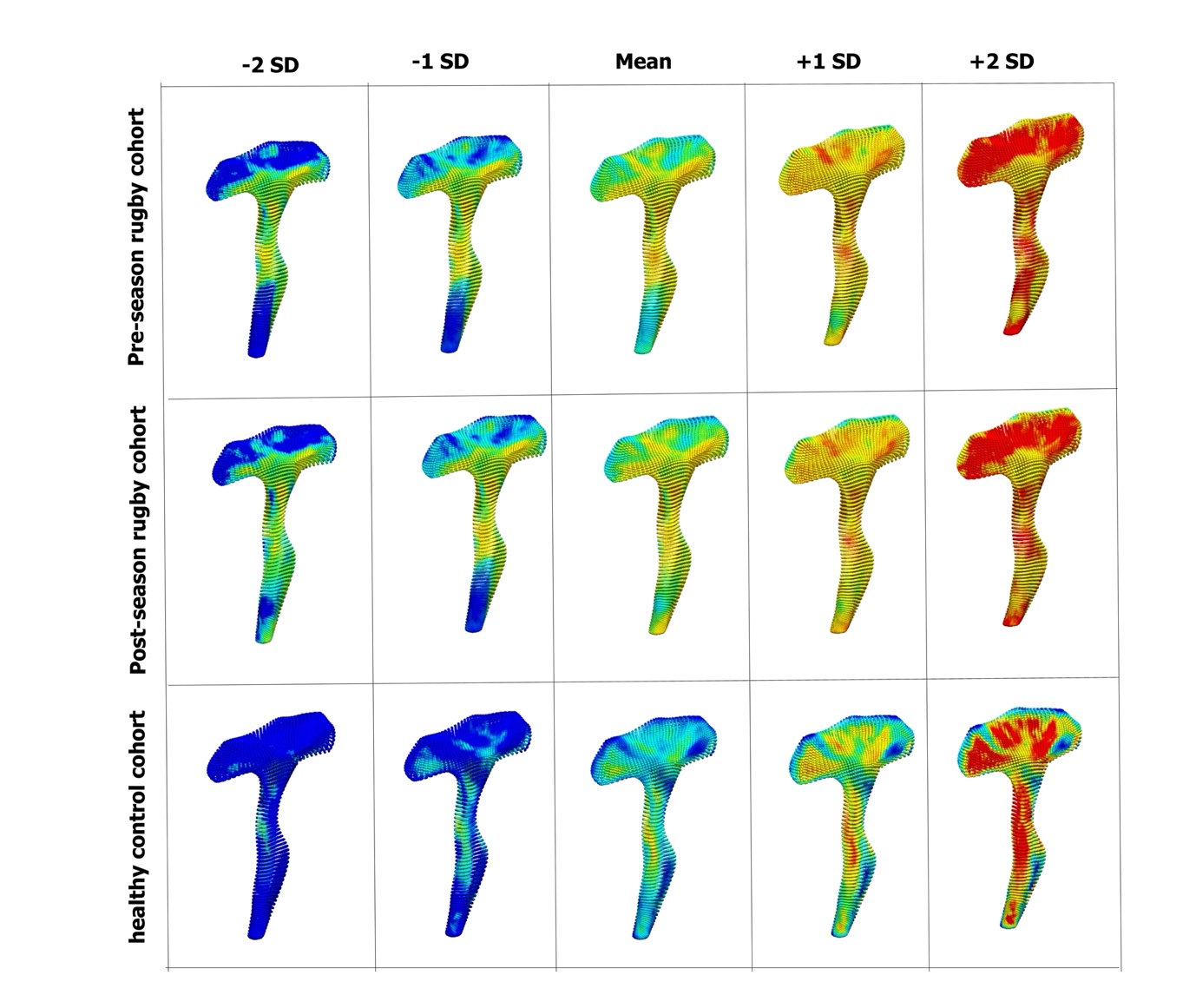
Supplementary Figure 2:** Pattern of AD value distribution in three different cohorts. The colour scale is the same as Figure 2 in the manuscript (0 ~ 0.00097)

**
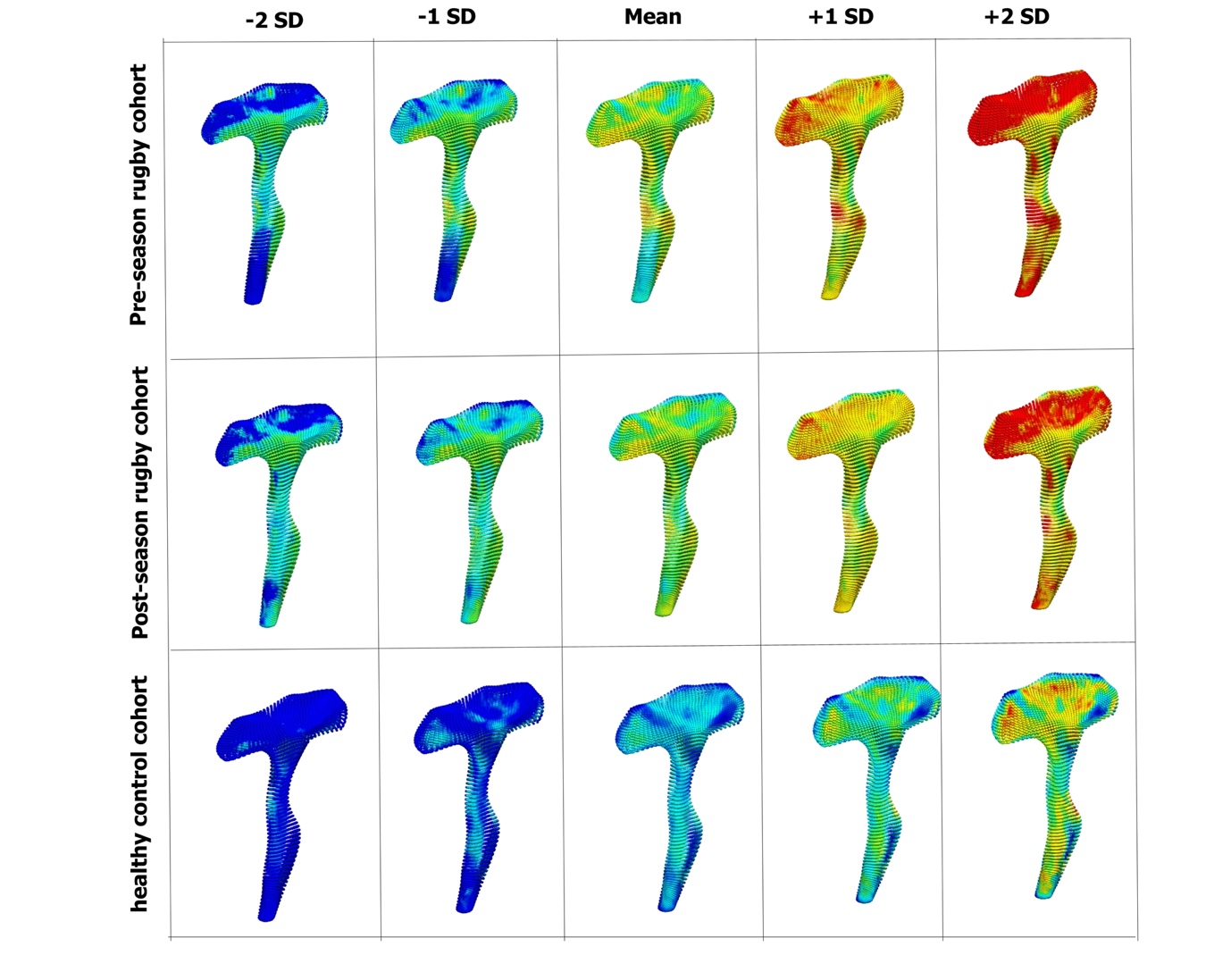
Supplementary Figure 3:** Pattern of RD value distribution in three different cohorts. The colour scale is the same as Figure 2 in the manuscript (0 ~ 0.00054)
